# Food Services Using Energy- and Protein-Fortified Meals to Assist Vulnerable Community-Residing Older Adults Meet Their Dietary Requirements and Maintain Good Health and Quality of Life: Findings from a Pilot Study

**DOI:** 10.3390/geriatrics3030060

**Published:** 2018-09-12

**Authors:** Tony Arjuna, Michelle Miller, Tomoko Ueno, Renuka Visvanathan, Kylie Lange, Stijn Soenen, Ian Chapman, Natalie Luscombe-Marsh

**Affiliations:** 1 National Health and Medical Research Council (NHMRC) Centre of Research Excellence in Translating Nutritional Science to Good Health, Discipline of Medicine, The University of Adelaide, Adelaide, SA 5005, Australia; tony.arjuna@ugm.ac.id (T.A.); renuka.visvanathan@adelaide.edu.au (R.V.); kylie.lange@adelaide.edu.au (K.L.); stijn.soenen@adelaide.edu.au (S.S.); ian.chapman@adelaide.edu.au (I.C.); 2 Department of Nutrition and Health, Faculty of Medicine, Universitas Gadjah Mada, Yogyakarta 55281, Indonesia; 3 Department of Nutrition and Dietetics, School of Health Sciences, Flinders University, Bedford Park, SA 5042, Australia; michelle.miller@flinders.edu.au (M.M.); tomoko.ueno001@gmail.com (T.U.); 4 Adelaide Geriatrics Training and Research with Aged Care (G-TRAC) Centre, National Health and Medical Research Council Centre of Research Excellence Frailty Trans-Disciplinary Research to Achieve Healthy Ageing, Discipline of Medicine, The University of Adelaide, Paradise, SA 5075, Australia; 5 Aged and Extended Care Services, The Queen Elizabeth Hospital, Central Adelaide Local Health Network, Woodville South, SA 5011, Australia; 6 Nutrition and Health Program, Health and Biosecurity Business Unit, Commonwealth Scientific Industrial Research Organisation (CSIRO), Adelaide, SA 5000, Australia

**Keywords:** elderly, meals on wheels, nutritional status, functional status, quality of life, hospital admission, human, meal services

## Abstract

The effects of “standard (STD)” vs. “protein- and energy-enriched (HEHP)” food-service meals on the nutrient intake, nutritional status, functional capacity, and wellbeing of older adults was investigated using a 12 week, double-blinded, parallel group design. All participants received dietetics counseling and either an STD (2.3 MJ and 30 g protein per meal) or a HEHP (4.6 MJ and 60 g protein) hot lunchtime meal for at least 3 days/week; those who did not want food-service meals were included in the control group (CON). Twenty-nine participants completed the study (STD = 7; HEHP = 12; CON = 10). From baseline to week 12, the HEHP subjects increased their mean daily energy intake from 6151 ± 376 kJ to 8228 ± 642 kJ (*p* = 0.002 for effect of time) and protein intake from 67 ± 4 g to 86 ± 8 g (*p* = 0.014 for effect of time). The MNA (Mini Nutritional Assessment) score was increased significantly in HEHP by 4.0 ± 1.1 points (*p* = 0.001), but not in the STD and CON groups (2.8 ± 2.1 points and 1.8 ± 1.1 points, *p* > 0.05). No difference was found for other clinical outcomes between the groups. The findings indicate that provision of HEHP-fortified food-service meals can increase energy and protein intake and improve the nutritional status of nutritionally at-risk older people.

## 1. Introduction

In Asia, Europe, and the United States, the prevalence of poor nutrition is high among hospitalized and institutionalized older people and is increasing in community-living adults aged 65 years and older [1,2]. Similar trends are occurring in Australia [3,4], with ~5%–10% of community-living older adults identified as being malnourished, and ~30%–40% ‘at risk’ of malnutrition [5,6]. Poor nutrition, particularly energy and protein malnutrition, has significant negative consequences including reduced muscle, cognitive and immune dysfunction, greater hospitalizations (number and length of stay), and premature entry into age-care homes [7,8]; all contribute substantially to increasing national healthcare expenditures across the world [9,10,11].

Access to affordable, high-quality nutrition that meets the requirements of older people is pivotal to the prevention and management of undernutrition in older people [11]. Meals-on-Wheels (MOW), a community health-service provider available in Australia, Canada, Ireland, the United Kingdom, and the United States, supports older people to live independently by providing healthy meals to their homes [12]. Ongoing government subsidization is pivotal for the financial viability of these food services and therefore it is important for them to demonstrate compliance with regulatory guidelines as well as the magnitude of their impact on the health and wellbeing of their clients [13]. While our group recently performed an audit of standard (STD) and protein- and energy-enriched (HEHP) meals from MOW South Australia Inc. (Adelaide, Australia) and demonstrated that the meals met the voluntary Home and Community Care (HACC) program guidelines and contributed to the daily energy and protein recommended daily intakes (RDIs) of older adults, a major limitation of that study was that we did not assess how the meals were actually being consumed by older adults [14]. To date, two separate Australian studies have reported that RDIs, including for protein, iron, calcium, thiamine and riboflavin, were not being achieved even though intakes were improved by the provision of standard MOW lunchtime meals; for example, 30%–45% of MOW clients still fell short of the RDI for protein despite consuming the meals [15,16].

Previously, we have shown that recipients, compared to nonrecipients of MOW in Australia, had fewer hospital admissions and reduced length of hospital stay over a 12-month period [17]. Whilst the two Australian studies have demonstrated that food services like Meals on Wheels can increase the nutritional intake of older adults, another study reported that significant improvements in body weight, and nutritional and functional status (over 6 months) for ‘at-risk’ and ‘malnourished’ older people were related, predominantly, to body mass index (BMI) and age, rather than to the nutrition being delivered by MOW in the form of a lunchtime meal [18]. The reasons why RDIs were not being met included splitting meals across the day, sharing meals, and large portion sizes that led to food wastage.

Accordingly, the aim of the study was to compare the effects of fortified and nonfortified hot lunchtime meals at least three days/week combined with standard dietetic counselling, compared to dietetic counselling only (no meal provided), on the primary outcomes of energy and protein intakes and nutritional status. Secondary outcomes, including physical capacity, general and psychological wellbeing, quality of life, and hospitalizations (number and duration of stay), and the level of satisfaction with meals and general service provided by the meal service, were also measured.

## 2. Materials and Methods

### 2.1. Participants

Individuals aged 70 years or greater and perceived to be ‘at risk’ of poor nutrition and in need of nutritional support were recruited from MOW South Australia Inc., via advertisements on community notice boards from general-practice clinics and local hospitals (particularly the dietetic departments); most individuals from the MOW South Australia Inc. database had been referred to that service within ≤3 months. Following referral, each individual was seen by a MOW client-assessment officer to explain the study, obtain written informed consent for their name and contact details to be forwarded to the university research team, and to answer several short questions to determine if they met three or more of the following criteria: (1) obviously underweight/frail; (2) reduced appetite; (3) unintentional weight loss over the past year; (4) unable to shop; (5) unable to prepare food for self; (6) is unable to feed self; (7) mouth or teeth swallowing problems; (8) obviously overweight, affecting quality of life; (9) unintentional weight gain over the past year; (10) special diet (Supplementary Table S1). Individuals identified as having three or more of these 10 eligibility criteria were referred to a member of the research team to determine full eligibility for the trial. Inclusion criteria included a Mini Nutritional Assessment (MNA) score > 17 ≤ 23.5 and/or a BMI < 24.0 kg/m^2^, plus having reported reduced appetite or unintentional weight loss over the past year and unable to shop or prepare food. Participants were excluded if they had a clinical diagnosis of dementia or significant depression, or were severely malnourished.

Individuals were allocated to the control group when they met the eligibility criteria but for personal reasons (i.e., received help with meals from family or friends, perceived themselves to be healthy, or refused assistance) did not want to receive support from MOW. Individuals were free to withdraw from the study at any time without affecting their ongoing or future relationship with MOW or the research team.

Ninety-five older individuals who had been in the MOW database for less than ~3 months and were only purchasing 1 to 2 meals weekly, or intermittently, were referred to the study. An additional 22 individuals were referred from other sectors of the community including the database of the Royal Adelaide Hospital Testosterone Study (i.e., *n* = 17) and self-referral (*n* = 5). Of these 117 individuals, 29 did not have 3 or more of the key criteria and 47 did not wish to participate for various personal reasons, including being too busy or waiting to be admitted to a hospital or nursing home.

### 2.2. Study Design

This pilot study had a 12-week double-blinded parallel-group design. The study was conducted between September 2012 and September 2013 at Flinders University and the University of Adelaide, Adelaide, Australia. MOW recipients were randomized to either the STD group or the HEHP group. In addition, there was a CON group that contained eligible participants who declined MOW services. Both the recipients of MOW services and the researchers were unaware of meal allocation to STD or HEHP, and participant randomization was performed by MOW staff who took no part in data collection or analyses. Meals were provided for at least 3 days per week. The Southern Adelaide Clinical Human Research Ethics Committee and the University of Adelaide Human Research Ethics Committee approved the study (Approval Number: 510.11). The trial was registered with the Australia and New Zealand Clinical Trial Registry (www.anzctr.org.au, registration number ACTRN12612000986875). All participants provided written informed consent prior to their inclusion.

### 2.3. Nutritional Intervention

At baseline prior to commencing the intervention, the STD, HEHP, and Control groups received 1 h of dietetic counselling from a qualified dietitian who estimated each individual’s energy requirements using the Schofield equation and other nutrient requirements based on Australian Nutrient Reference values [19,20]. Individuals were given strategies on how to achieve their RDIs.

In addition to basic dietetic counselling and monitoring, participants who were randomized to either STD or HEHP received MOW meals that were packed at a commercial kitchen facility located at Kent Town, Adelaide and that had been cooked by 1 of 3 trained chefs who were full-time employees of MOW South Australia Inc. All meals represented the typical 3-course hot lunchtime meal provided by MOW South Australia Inc., i.e., each lunchtime meal included a soup, a main dish, and a dessert. Recipes for the HEHP main course remained largely the same as for the STD main course; the energy and protein content of the STD meal was manipulated by fortifying the recipes of the soups and desserts with skim-milk powder, Beneprotein, Sustagen Hospital Formula (supplied from Nestlé Australia Ltd., New South Wales, Australia), cream, custard, and extra cheese, margarine, or oil incorporated for the gravies/sauces. Participants consumed their usual home-prepared food for other meal times throughout the day. The final energy and protein content of the prescribed STD meals was ~2.3 MJ and 30 g protein, while the prescribed HEHP meals contained 4.6 MJ and 60 g protein; hence, the STD contained ~33% and HEHP meals contained ~66% of estimated daily energy and protein requirements.

### 2.4. Outcome Assessment Methods

All outcomes were determined at baseline and week 12 by a trained research dietitian, and all measurements were performed in the homes of participants.

Energy and macronutrient intake, determined at baseline and week 12 using multipass dietary recalls (i.e., recalls over 2 weekdays and 1 weekend day), which were conducted with each participant as a face-to-face interview by a trained dietitian [21,22].

Nutritional status was determined using indices of: (1) BMI, in which body weight was measured using Tanita digital scales (Model BF-679 W, Western Australia, Australia) while participants wore light clothing and no shoes and height was measured using a portable stadiometer (Seca 213 Potable Stadiometer, Seca, CA, USA); (2) skinfold at midarm; (3) the circumferences of midarm and calf; (4) MNA (a score of <17 indicates that the participant is malnourished, 17–23.5: at risk of malnutrition, >23.5: well-nourished); and (5) the Simplified Nutritional Appetite Questionnaire (SNAQ; a score of ≤14: significant risk of ≥5% weight loss within 6 months, >14: no risk of weight loss) [23,24]. Physical capacity was determined from the following indices: (1) handgrip strength test using a calibrated dynamometer (Jamar Dynamometer, IL, USA); and (2) gait speed measured using the self-paced 3-meter walk test [25,26,27]. Both anthropometric and physical-capacity measurements were performed in triplicate and the average of the three values represented the final values used in the analysis.

General wellbeing and quality of life over the last week were determined using the Hawthorne Quality of Life questionnaire (AQoL version 1; continuous scale between 0 and 45 (unweighted scoring), with lower scores indicating better quality of life). Psychological wellbeing was determined using the Geriatric Depression Scale questionnaire (GDS; a score of >5: suggestive of depression, >9: almost always depression) [28,29].

Hospital admission, length of hospital stay (LOS), frequency of falls over the last 3 months were self-reported by participants and/or their family at time of incidence; level of satisfaction with meals and general service provided by MOW, including strengths and weaknesses, were determined using an anonymous survey at week 12.

### 2.5. Data Analysis

The dietary recalls were analyzed using FoodWorks version 6.2 (Xyris Software, Highgate Hill, Queensland, Australia) and the Australian nutrient composition database [30]. Nutrient contents of the STD and HEHP meals were calculated based on recipes provided by the MOW South Australia Inc. In addition, where participants consumed homemade meals or snacks, the nutrition-information panels from all food products/ingredients used within the recipe were used to estimate the nutrient composition per serve. For each participant, measurement of each daily nutrient intake was expressed as a percentage of their RDIs. For each question in the meal and service survey, the frequency of respondents to each answer was determined.

One-way analysis of variance (ANOVA) and independent-sample t-tests were used to compare differences in all baseline characteristics, and to determine differences between participants classified as completers (as per protocol) and noncompleters.

There were 30% (*n* = 12) of participants who did not complete the 12 week pilot study as per protocol, and therefore intention-to-treat (ITT) analysis using multiple imputation (using 20 imputations for all outcomes) was used to determine the effect of group on the change from baseline to week 12 for all outcomes; this type of analysis reduces bias that occurs if only completers’ data were analyzed and provides a more reliable estimate of true effect size [31]. Furthermore, compared to more traditional methods, such as complete case analysis, ITT analysis using the last observation carried forward, or more modern maximum likelihood-based methods, recent studies have reported that multiple imputation showed superior performance, smaller bias, and increased the study power, particularly in pretest—post-test study design such as ours [32,33]. Hence, in the current study, we derived a multiple imputation model for each nutritional outcome and it was based on identification of: (1) baseline variables that were associated with the probability of missing data at week 12; (2) variables that were associated with the week 12 outcome amongst participants with observed data; and (3) variables predefined to be included in the analysis model. Continuous nutrient and clinical outcomes were analyzed using analysis of covariance (ANCOVA) of the change from baseline to week 12 values with fixed effects for treatment group, age, gender, baseline energy, and the baseline value of the corresponding outcome. Assumptions of normality, variance, and homogeneity of slopes were assessed for all models. Pairwise contrasts, adjusted for multiple comparisons by Tukey HSD, were used to test for a difference between each intervention group and control (i.e., STD vs. CON, and HEHP vs. CON). Frequency of hospital admissions, length of hospital stay, and frequency of falls were categorized and analyzed using ordinal regression and included the same confounders as listed above. All statistical analyses were performed by SPSS (v.21.0 for Windows, IBM Corp, Armonk, NY, USA).

The data presented in the results section represent the unadjusted means and standard error of the means (SEMs) from multiple imputation models, but it is important to note that we found consistent and comparable levels of statistical significance from both completers as per protocol and ITT analyses.

## 3. Results

### 3.1. Participants

**Figure 1:**
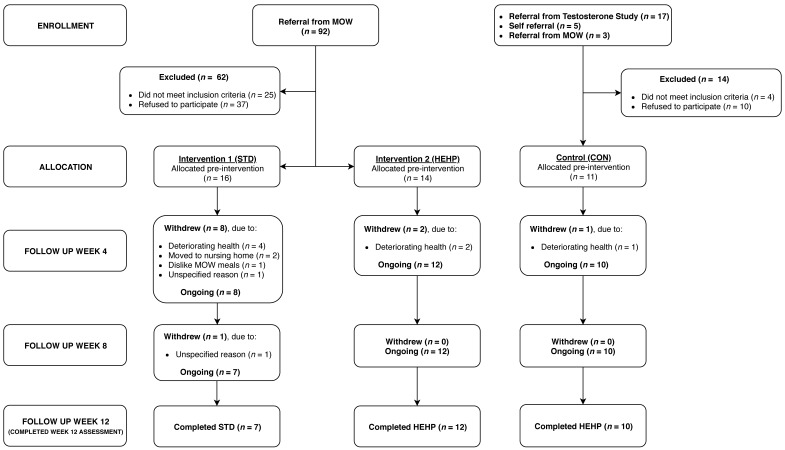
Flowchart of participants through the study.

Forty-one adults commenced the 12-week study (STD = 16; HEHP = 14; CON = 11) and 29 participants completed the study (STD = 7; HEHP = 12; CON = 10). The number of participants initially allocated to STD and HEHP was unbalanced because a husband and wife were enrolled and randomized to STD but, approximately one week after randomization, it was discovered that the husband was ineligible as he was moving into a nursing home. Reasons for withdrawal were comparable between STD and HEHP groups (Figure 1). There were no differences in baseline characteristics between the STD, HEHP, or CON groups (Supplementary Table S2, *p* > 0.05), or between the completers and noncompleters (Table 1, *p* > 0.05).

### 3.2. Nutrient Intakes

The HEHP group had a significantly increased energy, protein, total fat, saturated fat, and carbohydrate intake from baseline to week 12, while no significant changes were found in the STD and CON groups (Table 2). In addition, for HEHP compared with CON, the magnitude of change from baseline to week 12 was greater for total fat (HEHP: 19 ± 8 vs. CON: −0.2 ± 6 g per day), saturated fat (HEHP: 9 ± 3 vs. CON: −2 ± 3 g per day), and sugar (HEHP: 11 ± 9 vs. CON: −5 ± 11 g per day) (all Ps for effect of treatment were <0.05; Table 2), whereas the change from baseline for each of these nutrients was not different between STD and CON (all Ps for effect of treatment were >0.05, Table 2).

**Figure 2:**
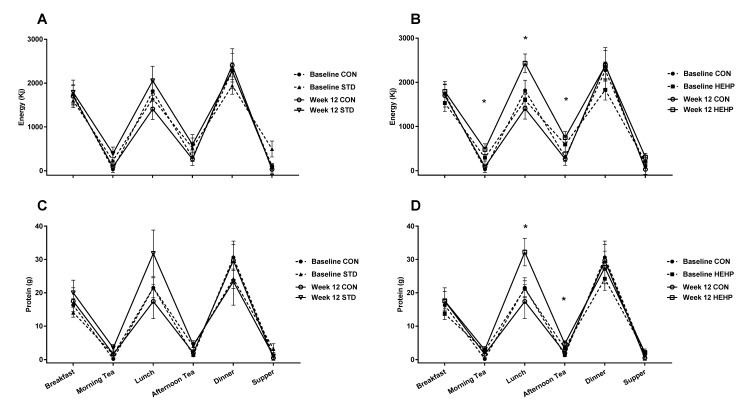
Patterns of energy and protein intake for (**A** and **C**) STD vs. CON and (**B** and **D**) HEHP vs. CON. Data presented as unadjusted mean energy and protein intakes at various eating occasions throughout the day at baseline and week 12. One-way ANOVA was used to compare differences in baseline parameters between the three groups and there were no significant differences between groups (all Ps > 0.05). ANCOVA of the week 12 data was performed using fixed effects for treatment group, age, gender, baseline energy, and the baseline value of the corresponding outcome; * Significant difference between HEHP and CON at week 12.

Figure 2 shows that, after 12 weeks of intervention, HEHP compared with CON reported greater energy intakes at morning tea, lunch, and afternoon tea, and protein intakes were greater at lunch and afternoon tea (***p*** < 0.05 for all time-by-group interactions). Figure 2 also highlights that, for both STD and CON, there was no significant increase in either energy or protein from baseline to week 12 at any eating occasion (all ***p*** > 0.05).

At week 12, the MOW meals provided the HEHP group with a significantly higher percentage of their RDI for energy (67 ± 16% vs. 39 ± 16%, ***p*** = 0.003) and protein (87 ± 21% vs. 51 ± 20%, ***p*** = 0.005) than the STD group. For overall intake throughout the day, all groups met their daily energy requirement and most of the nutrient requirements at baseline and week 12, except for calcium and fiber (Table 3). The percentage of RDIs achieved during this intervention for micro minerals or trace elements could not be determined using either the multiple imputation model or the completers’ analysis because there was a large degree of variability in those data.

### 3.3. Clinical Outcomes

Table 4 shows that there was a significant increase in the MNA score from baseline to week 12 in HEHP, but not the STD and CON groups. Additionally, triceps-skinfold thickness was significantly reduced in CON, but not the HEHP and STD groups. There was no difference between any of the groups for the changes from baseline to week 12 for the markers of nutritional status, physical function, quality of life, or psychological wellbeing (all *p* for effect of treatment were >0.05). Table 5 also shows that, by week 12, there was no statistically significant difference between groups for the number or length of stay for hospital admissions, or the number of self-reported falls (all *p* for effect of treatment were >0.05).

### 3.4. Surveys

At week 12, ~50% of the 19 participants receiving STD and HEHP meals were either ‘very satisfied’ or ‘satisfied’ with the meals, while 16% were ‘unsure’ and 33% ‘dissatisfied’ with the meals. Additionally, 83% of participants were ‘very satisfied’ and 17% were ‘satisfied’ with the overall service provided by MOW and staff/volunteers who delivered the meal. Furthermore, when asked about the affordability of MOW meals and services, more than half (58.5%) of the elderly ‘strongly agree’ and 31.7% ‘agree’ that MOW meals and services are affordable. Some notable comments from the elderly: “As a new member, I am very pleased with the quantity and standard of food supplied for $7 daily, and the quality of service is excellent”.

“Enjoying MOW because of less preparation for food.”

“Meals are affordable if they are actually eaten.”

When all participants who completed the study were asked about their nutritional adequacy, nearly 50% perceived themselves as being ‘nutritionally adequate’ despite the fact that they all had an MNA score ≤ 23.5, indicating they were at risk of malnutrition; there were more women than men (29% vs. 18%) who felt their nutritional status was not good. Although the majority of participants who received meals rated portion size of their meal as ‘just right’, 30% rated the portion size as ‘too much’ to consume at lunch (particularly those in the HEHP group (*n* = 5 vs. 1)) and therefore reported on eating their soup and/or dessert on another occasion during the day. Reasons for accepting or declining MOW services appear to relate to the fact that the majority of the elderly (64%) were receiving shopping assistance, particularly older women (37.7% compared to 26.7% among men). Furthermore, about a third of the older adults receive support for meal preparation and cooking from family members, carers, or friends, and also use some types of nutritional supplements (e.g., Milo^®^ and Sustagen^®^).

## 4. Discussion

Our findings indicate that an energy- and protein-enhanced meal from food-service providers like MOW South Australia Inc. significantly increased mean daily energy and macronutrient intakes and MNA scores after 12 weeks, whereas neither the CON nor STD program increased these intakes over 12 weeks. However, despite these improved intakes, there was no significant differential effect of either type of meals when compared with the CON, on the other markers of physical capacity, general and psychological wellbeing, quality of life, or hospitalizations. This is mainly due to the fact that participants in all groups were not markedly malnourished, as evident from good nutritional intake being observed at baseline. It is likely that older people who have poorer intake and lower BMI than participants of this study would likely see greater benefit from the intervention. Our findings indicate that energy- and protein-enhanced meals are an effective strategy to help vulnerable older adults aged 70 years and older to improve nutritional status and achieve their RDIs, especially for energy, protein, carbohydrates, and total fat.

The magnitude of change in nutrient intakes reported here is consistent with findings from previous studies [18,34,35], and especially those that have measured pre- and post-intervention intakes from consumers compared with nonconsumers of MOW, assessed over eight weeks [36,37]. For example, a quasiexperimental study by Roy and Payette reported mean daily energy and protein intakes were increased by 10% and 16% (and provided on average 119% of RDI for protein) among frail consumers of MOW meals compared with nonconsumers whose intakes remained stable [36]; this is comparable to the improvement observed within the HEHP group for mean daily energy but a notable difference was that our participants were already consuming more than 100% of their RDIs at baseline (i.e., in our study, mean daily intakes of both energy and protein increased by 10% from baseline and represented 116 ± 17% and 188 ± 37% of RDI by week 12). Similarly, a recent eight-week observational study conducted in the United States of 51 older home-delivered-meal clients (mean age of 74.11 years, 58% at risk of malnutrition and 34% malnourished) indicated that mean daily energy intake was substantially and significantly increased from 5.7 to 6.8 MJ/day, and that mean protein intake was increased from 54.1 to 73.7 g/day [37]. However, a point of difference between the study by Wright et al. and our study again highlights that individuals receiving MOW in the United States reportedly have mean baseline energy and protein intakes substantially lower than in the present study for either the STD and HEHP groups; instead, U.S. recipients of MOW reported intakes that were comparable with our CON group. In addition to differences in nutrient intakes at baseline, 34% of the participants in the study by Wright et al. [33] were malnourished and, therefore, were probably less likely to be able to eat all the food provided, or more likely to eat less at other meals due to increased sensitivity to the gastrointestinal and hedonic effects of the nutrients [38,39]. Our survey data also highlighted that older people struggled to eat a large meal in one sitting; 30% stated they ate the soup and dessert for afternoon tea or dinner. In fact, ~33% of our participants in both the STD and HEHP groups, were supplementing their intakes with other foods, including sandwiches, Milo^®^, milk, Sustagen^®^, and fruit, suggesting that the provision of smaller meals and/or snacks from MOW services may be warranted to decrease older adults feeling overwhelmed by large meal sizes and experiencing feelings of guilt that they cannot eat it all, or that it is not affordable because the food spoils before eat it. However, a separate Australian pilot study has reported that not all MOW clients at risk of malnutrition perceived the snacks to be beneficial to them after four weeks and concluded other strategies to improve the nutritional and health status of older adults may be more appropriate [34].

While our findings demonstrate that the CON, STD, and HEHP, groups had comparable effects on maintaining markers of nutritional status, it should be noted that the HEHP group did experience significant increases in MNA scores, and (albeit not significant) body weight that, if sustained, may confer a clinical benefit—i.e., total MNA score was increased by 4.0 ± 1.1 points (*p* = 0.001) and body weight was increased by 1.1 ± 1.4 kg with HEHP, whereas by these outcomes were increased by 2.8 ± 2.1 points and 0.8 ± 1.3 kg with STD, and by 1.8 ± 1.1 points and by 0.1 ± 1.0 kg with CON.

Moreover, we found that markers of physical capacity, general and psychological wellbeing, and quality of life remained stable (i.e., they neither improved, nor declined, over the 12-week study) with all treatments. While there is some evidence that the benefits of increased protein intake are only apparent when ~20–30 g of protein is consumed at each main meal, three times per day, or as a protein- and energy-fortified nutritional liquid supplement [40,41], rather than as a large dose at a single meal [42], it is highly likely that comparable findings between the groups in this study were due, at least in part, to a number of reasons, including: (i) participants in all groups adopting the dietitian’s advice of consuming foods that were higher in energy and protein at both their main and midmeal eating occasions (Figure 2); (ii) that all participants were already exceeding their RDIs for both energy and protein at baseline—in fact, protein intakes at baseline for all three groups were already reaching levels of 1.2–1.4 g/kg/day, which is the new level being recommended for older adults by several international expert working committees [42]; and/or (iii) that for some individuals, a critical nutritional deficiency was not actually overcome (i.e., some individuals, despite reportedly consuming high intakes, are suffering malabsorption issues). Regardless of reason(s), these comparable findings demonstrate dietetic counseling should be an additional offering provided by food-service providers (including MOW South Australia Inc.) given that some older adults and/or their carers require (and value) support from trained health professionals to increase their health knowledge and periodically assess their health and wellbeing.

A recent review has noted that a major limitation of previous research in this area is that many studies do not use random allocation of treatments and/or do not have a control group [43]. Hence, major strengths of this study were that it used a randomization design to allocate the two meal interventions and it included a control group. Our control consisted of no provision of MOW meals throughout the 12 weeks, plus a one-hour session of dietetics counseling at baseline; dietetic counseling was included to ensure all participants, even the controls who were ‘at risk’, were as educated about the consequences of malnutrition and how energy and protein rich foods may help mitigate them. However, our data should be viewed circumspectly because it is likely that, over a longer timeframe, dietetics counseling in addition to provision of nutritious meals would have the greatest impact on nutritional status and, hence, health. For example, a nonrandomized intervention study of 355 community-living participants (aged 76.7 ± 3.2 years) demonstrated that nutrition education and counseling, when combined with the provision of meals in either a dining hall or home setting, not only improved nutrition risk scores, but also resulted in more participants “eating two or more meals per day” in home-delivered meals (76% to 81.6%), or “consuming five or more servings of fruit and vegetables” (38% to 41.4%), and reducing “more than three servings of alcohol drinking” in congregate meals group (8.4% to 4.8%) [44].

This study had several limitations. Recruitment of participants was only from the Adelaide region of Australia, and the exclusion of individuals diagnosed with dementia and/or depression meant that we ended the pilot research with a small “final” sample size and a cohort who were possibly less sensitive to the effects of treatment. While we had a total of 117 referrals through MOW South Australia Inc. and 25 through other recruitment channels, only 35% of all referrals commenced the study and only 25% completed it. This demonstrates that many community-living older adults perceive their nutritional and health status to be better than it actually is; this notion was also supported by participants’ responses to the survey questions. In addition, ~21% of our eligible and allocated participants withdrew from the study within the first few weeks due to deteriorating health or entry into a nursing home, while another 5% withdrew without giving a reason or because they did not like the meal. Reasons for withdrawal at least concur with reasons previously reported by Choi et al., who found that more than 25% withdrew from the MOW food service within the first few weeks due to prolonged hospitalization or placement to nursing home, 15% due to improved health, and 15% due to dissatisfaction with MOW meals [45]. However, despite using intention-to-treat (based on a customized multiple imputation) analyses for each outcome, it is likely that the 30% dropout rate in the first weeks has caused some bias, particularly for specific micronutrients/trace elements and the number of hospital admissions, length of hospital stay, and falls.

## 5. Conclusions

This study showed that the HEHP food-service meals increased nutrient intake and improved nutritional status in community-dwelling older people at risk of malnutrition, whereas the control and standard meals did not have this effect. However, there was no significant effect of either type of meal when compared with the CON on the other markers of physical capacity, general and psychological wellbeing, quality of life, or hospitalizations, possibly related to the good nutrient intakes and BMIs of all subjects groups at baseline, and the relatively low subject numbers. Future studies are indicated of older people with poorer intakes and nutritional status than those in this study. Research of a longer duration and with larger numbers of recipients using food services like MOW South Australia Inc. are also needed to determine the cost effectiveness of different types of meals.

## Figures and Tables

**Table 1:** Baseline characteristics of the total participants, completers and non-completers ^^^*.

Characteristics	Total Enrolled	Completers	Non-Completers
	(*n* = 41)	(*n* = 29)	(*n* = 12)
Male/Female (*n*)	19/22	13/16	6/6
Age (year)	83.9 ± 0.9	83.1 ± 1.1	85.7 ± 1.9
Height (m)	1.63 ± 0.09	1.63 ± 0.01	1.63 ± 0.03
Body weight (kg)	58.0 ± 1.6	57.3 ± 1.7	59.8 ± 3.6
Body mass index (BMI) (kg/m^2^)	21.9 ± 0.6	21.7 ± 0.7	22.2 ± 1.0
Self-reported percent weight loss in the previous three months	4.0 ± 1.0	4.9 ± 1.3	1.9 ± 1.0
Number of medications	7.1 ± 0.6	7.2 ± 0.8	7.0 ± 0.7
Multivitamins/minerals	2.0 ± 0.3	2.2 ± 0.4	1.2 ± 0.3
Number of unmet needs based on Meals on Wheels (MOW) assessment	3.7 ± 0.2	3.7 ± 0.2	3.6 ± 0.4
Living status (*n*):			
Alone	26	17	9
With significant other	15	12	3
Require nutrition support (*n*)			
Yes	14	10	4
No	27	19	8
MOW referral source (*n*)			
Self	17	6	1
Health professional	20	3	4
Family	27	7	3
Hospital	23	5	3
Doctor	13	3	1
Friends	0	1	0
Others	0	4	0

Data presented as unadjusted mean ± SEM or *n*; ^^^ one-way analysis of variance (ANOVA) was used to compare differences between completers and dropouts for all baseline characteristics; * there were no significant differences between completers vs. non-completers, for any of the baseline characteristics (all *p* > 0.05).

**Table 2:** Total daily nutrient intakes at baseline and change from baseline to week 12 for STD, HEHP, and CON ^^#^.

Nutrients	STD		HEHP		CON		*p* Value Change from Baseline for STD ^†^	*p* Value Change from Baseline for HEHP ^†^	*p* Value Change from Baseline for CON ^†^	*p* Value STD vs. CON ^‡^	*p* Value HEHP vs. CON ^‡^
	Baseline (*n* = 16)	Change from Baseline (*n* = 16)	Baseline (*n* = 14)	Change from Baseline (*n* = 14)	Baseline (*n* = 11)	Change from Baseline (*n* = 11)					
Energy (kJ)	6512 ± 376	635 ± 979	6151 ± 376	1958 ± 621	6278 ± 468	211 ± 422	0.52	0.002 *	0.62	0.52	0.06
Protein (g)	68 ± 6	17 ± 17	67 ± 4	18 ± 7	71 ± 5	−0.5 ± 8	0.32	0.014 *	0.18	0.61	0.34
Total fat (g)	55 ± 5	6 ± 12	51 ± 5	19 ± 8	51. ± 4	−0.2 ± 6	0.62	0.018 *	0.97	0.26	0.021 *
Saturated fat (g)	22 ± 2	3 ± 4	21 ± 2	9 ± 3	23 ± 2	−2 ± 3	0.50	0.007 *	0.42	0.15	0.004 *
Carbohydrate (g)	196 ± 17	−5 ± 29	180 ± 11	43 ± 18	173± 12	6 ± 13	0.85	0.021 *	0.63	0.81	0.09
Sugars (g)	97 ± 12	−9 ± 16	100 ± 11	11 ± 9	85 ± 9	−5 ± 11	0.56	0.20	0.66	0.61	0.049 *
Fiber (g)	17 ± 2	−0.1 ± 8	17 ± 2	2 ± 3	19 ± 2	5 ± 5	0.98	0.53	0.27	0.32	0.52
Vitamin C (mg)	75 ± 15	3 ± 30	74 ± 12	10 ± 19	77± 16	−2 ± 13	0.93	0.59	0.90	0.84	0.65
Calcium (mg)	824 ± 101	200 ± 211	887 ± 68	181 ± 98	801 ± 110	−32 ± 107	0.35	0.07	0.76	0.41	0.17
Iron (mg)	10 ± 1	−0.4 ± 2	7 ± 1	2 ± 1	10 ± 1	−0.5 ± 1	0.84	0.14	0.70	0.71	0.66

Data presented as unadjusted mean ± SEM derived from intention-to-treat analysis using multiple imputation (i.e., 20 imputations per outcome), which reduces the bias of using completers-only data; ^^^ one-way ANOVA was used to compare differences in baseline parameters between the three groups, and there were no significant differences between groups (all ***p*** > 0.05); ^#^ analysis of covariance (ANCOVA) of the change from baseline to week 12 data was performed using fixed effects for treatment group, age, gender, baseline energy, and the baseline value of the corresponding outcomes; ^†^ ***p*** values from paired t-test of baseline and week 12; **^‡^** ***p*** values from adjusted ANCOVA test; * significant difference ***p*** < 0.05; STD: standard group, HEHP: high energy- and protein-fortified group, CON: control group.

**Table 3:** Percentage of Recommended Dietary Intake achieved at baseline and week 12 STD, HEHP and CON ^^^.

Nutrients	STD		HEHP		CON	
	Baseline (*n* = 16)	Week 12 (*n* = 16)	Baseline (*n* = 14)	Week 12 (*n* = 14)	Baseline (*n* = 11)	Week 12 (*n* = 11)
Energy ^#^	106	116	102	132	102	106
Protein ^†^	102	125	103	124	103	102
Total fat	91	94	89	93	88	82
Saturated fat	130	138	130	140	141	115
Carbohydrate	112	98	109	102	103	105
Sugars	108	99	111	122	94	89
Fiber	68	67	68	77	74	95
Vitamin C	166	176	163	190	171	159
Calcium	63	76	68	82	62	62
Iron	119	113	108	133	126	123

Data presented as % of recommended daily intakes (RDIs); ^^^ based on Australian Nutrient Reference values; ^#^ based on individual energy requirement calculated using Schofield equation; ^†^ based on individual protein requirement of 1.2 g/kg body weight.

**Table 4:** Clinical outcomes at baseline and week 12 for STD, HEHP, and CON ^^#^.

Clinical Outcomes	STD		HEHP		CON		*p* Value Change from Baseline for STD ^†^	*p* Value Change from Baseline for HEHP ^†^	*p* Value Change from Baseline for CON ^†^	*p* Value STD vs. CON ^‡^	*p* Value HEHP vs. CON ^‡^
	Baseline (*n* = 16)	Change from Baseline (*n* = 16)	Baseline (*n* = 14)	Change from Baseline (*n* = 14)	Baseline (*n* = 11)	Change from Baseline (*n* = 11)					
MNA score	19.6 ± 0.5	2.8 ± 2.1	18.6 ± 1.2	4.0 ± 1.1	22.0 ± 0.8	1.8 ± 1.1	0.18	0.001 *	0.10	0.83	0.65
SNAQ score	12.7 ± 0.6	1.5 ± 1.1	12.2 ± 0.6	0.9 ± 0.5	13.9 ± 0.6	0.5 ± 0.6	0.18	0.09	0.38	0.89	0.65
Body weight (kg)	58.9 ± 2.9	0.8 ± 1.3	57.4 ± 3.0	1.1 ± 1.4	57.6 ± 2.0	0.1 ± 1.0	0.56	0.44	0.94	0.28	0.37
BMI (kg/m^2^)	22.2 ± 0.8	0.8 ± 0.6	21.9 ± 1.3	0.3 ± 0.5	21.4 ± 0.7	0 ± 0.4	0.15	0.58	0.94	0.15	0.46
Calf circumference (cm)	32.1 ± 0.8	0.7 ± 1.1	32.0 ± 0.9	0.6 ± 0.5	32.5 ± 0.7	−0.2 ± 0.3	0.52	0.25	0.61	0.45	0.46
Arm circumference (cm)	24.0 ± 0.8	1.6 ± 1.0	25.1 ± 1.1	0 ± 0.7	25.2 ± 0.7	0 ± 0.5	0.09	0.97	0.98	0.14	0.92
Triceps skinfold (mm)	8.0 ± 1.9	−0.9 ± 1.2	7.9 ± 1.3	−2.0 ± 1.3	9.7 ± 1.5	−1.4 ± 0.7	0.46	0.14	0.032 *	0.66	0.29
Handgrip strength (kg)	19.5 ± 1.8	0.0 ± 1.7	16.7 ± 1.8	0.4 ± 0.9	21.8 ± 1.8	0.2 ± 1.1	0.99	0.64	0.82	0.97	0.98
Gait speed (m/s)	0.62 ± 0.07	0.06 ± 0.09	0.66 ± 0.07	−0.03 ± 0.05	0.74 ± 0.07	0.05 ± 0.08	0.49	0.51	0.55	0.86	0.24
AQoL	29.7 ± 1.5	0.6 ± 1.5	30.8 ± 1.4	0 ± 1.0	30 ± 1.5	−1.0 ± 1.4	0.72	0.97	0.51	0.59	0.65
GDS	5.5 ± 0.8	0.8 ± 1.2	4.7 ± 0.6	−0.7 ± 0.8	3.9 ± 1.2	0.8 ± 1.0	0.53	0.40	0.44	0.73	0.43

Data presented as unadjusted mean ± SEM derived from intention-to-treat analysis using multiple imputation (i.e., 20 imputations per outcome), which reduces the bias of using completers-only data; ^^^ one-way ANOVA was used to compare differences in baseline parameters between the three groups and there were no significant differences between groups (all *p* > 0.05); ^#^ ANCOVA of the change from baseline to week 12 data was performed using fixed effects for treatment group, age, gender, baseline energy, and the baseline value of the corresponding outcome; ^†^ *p* values from paired t-test of baseline and week 12; **^‡^** *p* values from adjusted ANCOVA test; * significant difference *p* < 0.05; MNA: Mini Nutritional Assessment; SNAQ: Simplified Nutritional Appetite Questionnaire; AQoL: Hawthorne Quality of Life questionnaire; GDS: Geriatric Depression Scale questionnaire.

**Table 5:** Hospitalization and number of falls at baseline and week 12 for STD, HEHP, and CON ^^#^.

Clinical Outcomes	STD		HEHP		CON		*p* Value Change from Baseline for STD ^†^	*p* Value Change from Baseline for HEHP ^†^	*p* Value Change from Baseline for CON ^†^	*p* Value STD vs. CON ^‡^	*p* Value HEHP vs. CON ^‡^
	Baseline (*n* = 16)	Week 12 (*n* = 16)	Baseline (*n* = 14)	Week 12 (*n* = 14)	Baseline (*n* = 11)	Week 12 (*n* = 11)					
Number of hospital admission (frequency)											
0 admissions	6	5	3	7	6	5	0.11	1.0	0.42	0.25	0.84
1 admission	8	3	9	3	3	2					
2 admissions	1	3	1	2	1	2					
3 or more admissions	1	5	1	2	1	2					
Total admissions	10	11	11	7	4	6					
Length of hospital stay (days)											
0 day	6	6	4	8	6	6	0.12	0.06	1.0	0.66	0.92
1–6 days	3	1	5	1	4	2					
7–13 days	4	0	2	2	0	1					
14 or more days	3	9	3	3	1	2					
Number of falls (frequency)											
0 falls	10	7	10	10	8	7	0.40	0.56	0.95	0.54	0.63
1 fall	5	5	2	3	1	2					
2 falls	0	4	1	1	2	2					
3 falls	1	0	1	0	0	0					

Data presented as unadjusted frequency derived from intention-to-treat analysis using multiple imputation (i.e., 20 imputations per outcome), which reduces the bias of using completers-only data; ^^^ one-way ANOVA was used to compare differences in baseline parameters between the three groups, and there were no significant differences between groups (all *p* > 0.05); ^#^ frequency of hospital admissions, length of hospital stay, and frequency of falls were categorized and analyzed using ordinal regression using fixed effects for treatment group, age, gender, baseline energy, and the baseline value of the corresponding outcome; ^†^ *p* values from paired t-test of baseline and week 12; **^‡^** *p* values from adjusted ANCOVA test; * significant difference *p* < 0.05.

## Supplementary Materials

The following are available online at http://www.mdpi.com/2308-3417/3/3/60/s1. Table S1: MOW Screening Questions; Table S2: Baseline characteristics of participants according to group allocation ^^#^.
