# Supplementary material for: Food Services Using Energy- and Protein-Fortified Meals to Assist Vulnerable Community-Residing Older Adults Meet Their Dietary Requirements and Maintain Good Health and Quality of Life: Findings from a Pilot Study
Source: Geriatrics (Basel). 2018 Sep 12;3(3):60. doi: 10.3390/geriatrics3030060 (PMC6319248; doi:10.3390/geriatrics3030060)
Supplement: Supplementary file 1 [file geriatrics-03-00060-s001.pdf]

Supplementary Table S1. MOW Screening Questions.

No.	Questions	YES	NO
1.	Obviously underweight/frail		
2.	Unintentional weight loss		
3.	Reduced appetite (food and fluid intake)		
4.	Mouth or teeth swallowing problems		
5.	Following special diet		
6.	Unable to shop for food		
7.	Unable to prepare other meals		
8.	Unable to feed self		
9.	Obviously overweight affecting quality of life		
10.	Unintentional weight gain		

Supplementary Table S2. Baseline characteristics of participants according to group allocation ^#.

Characteristics	STD (*n* = 16)	HEHP (*n* = 14)	Control (*n* = 11)	Total (*n* = 41)
Gender:				
Male/Female (n)	8/8	6/8	5/6	19/22
Age (year)	85.6 ± 1.7	83.8 ± 1.4	81.5 ± 1.6	83.9 ± 0.9
Height (m)	1.63 ± 0.1	1.62 ± 0.09	1.64 ± 0.06	1.63 ± 0.09
Body weight (kg)	58.9 ± 2.9	57.4 ± 3.0	57.6 ± 2.0	58.0 ± 1.6
BMI (kg/m^2)	22.2 ± 0.8	21.9 ± 1.3	21.4 ± 0.7	21.9 ± 0.6
Self-reported weight loss percentage in the previous three months	3.8 ± 1.4	5.9 ± 1.9	2.1 ± 1.9	4.0 ± 1.0
Number of medications	7.1 ± 0.8	6.1 ± 1.1	8.6 ± 1.2	7.1 ± 0.6
Nutritional supplements (Multivitamins / minerals)	2.0 ± 0.7	2.0 ± 0.5	2.0 ± 0.5	2.0 ± 0.3
Number of meals ordered from MOW	5.3 ± 0.3	5.0 ± 0.7	N/A	5.0 ± 0.2
Number of unmet needs based on MOW assessment	3.8 ± 0.3	3.6 ± 0.3	N/A	3.7 ± 0.2
Living status (*n*):				
1. Alone	9	12	5	26
2. With significant other	7	2	6	15
Oral nutrition support (*n*)				
1. Yes	5	7	2	14
2. No	11	7	9	27
MOW referral source (*n*)				
1. Self	3	2	N/A	17
2. Health professional	3	3		20
3. Family	4	4		27
4. Hospital	4	3		23
5. Doctor	2	2		13
6. Friends	0	0		0
7. Others	0	0		0

^ One-way ANOVA was used to compare differences in baseline characteristics between the three groups and completers against dropouts; # data presented as: mean ± SEM; N/A: not applicable.
